# Maternal reasons for requesting planned cesarean section in Norway: a qualitative study

**DOI:** 10.1186/s12884-019-2250-6

**Published:** 2019-03-29

**Authors:** Kristiane Tislevoll Eide, Nils-Halvdan Morken, Kristine Bærøe

**Affiliations:** 1 Department of Global Public Health and Primary Care, University of Bergen, Kalfarveien 31, 5018 Bergen, Norway; 2 Department of Obstetrics and Gynecology, Haukeland University Hospital, Jonas Lies veg 87, 5021 Bergen, Norway; 3 Department of Clinical Science, University of Bergen, Jonas Lies veg 87, 5021 Bergen, Norway

**Keywords:** Cesarean section, Maternal request, Qualitative methods, Fear of birth, Traumatic birth experience

## Abstract

**Background:**

Pregnant women who request a cesarean section in the absence of obstetric indication have become a highly debated issue in academic as well as popular literature. In order to find adequate, targeted treatment and preventive strategies, we need a better understanding of this phenomenon. The aim of this study is to provide a qualitative exploration of maternal requests for a planned cesarean section in Norway, in the absence of obstetric indications.

**Methods:**

A descriptive qualitative study was conducted consisting of 17 semi-structured, in-depth interviews with women requesting cesarean section and six focus group discussions with 20 caregivers (nine midwives, 11 obstetricians) working at a university hospital in Norway. Data were analyzed with Systematic Text Condensation, a method for thematic cross-case analysis.

**Results:**

Fear of birth emerged most commonly as a result of a previous traumatic birth experience that prompted a preference for a planned cesarean to avoid a repetition of the trauma. For some women in our study, postnatal care and the puerperal period were their crucial past experiences, and giving birth by planned cesarean was seen as a way to ensure mental rather than physical capability to care for the expected child after birth. Others were under the impression of being at high risk for an emergency C-section, and requesting a planned one was based on their perceived risk. Such perceptions included having a narrow pelvis, hereditary factors or previous birth outcomes. Some primiparas requested a planned cesarean based on a deep-seated fear since their early teens, accompanied by alienation towards the idea of giving birth. Some obstetricians participating in our study also experienced requests that lacked what they regarded as any well-grounded reason or significant fear.

**Conclusions:**

Behind a maternal request for a planned cesarean section are various rationales and life experiences needing carefully targeted attention and health care. Previous births are an important driver; thus, maternally requested cesareans should be regarded partly as an iatrogenic problem.

**Electronic supplementary material:**

The online version of this article (10.1186/s12884-019-2250-6) contains supplementary material, which is available to authorized users.

## Background

Rising cesarean section (CS) rates in high and middle income countries over recent decades have initiated concern about the overuse of CS [1]. Nordic countries have made a remarkable effort in keeping rates low [2]. Nevertheless, maternally requested surgery remains a controversial issue in academic and public debate.

CS on maternal request (CSMR) is defined as a planned CS conducted on maternal request when there is no obstetric contraindication for vaginal delivery [3]. There is a lack of explicit medical classification and secondary diagnoses are frequent, creating uncertainty of prevalence estimates [4]. A study from Norway found that 10% of CSs undertaken in its study period (1998–99) were conducted on maternal request, representing less than 1% of all births in Norway at that time [5]. This coincides with self-reported numbers (0.8%) by Fuglenes et al. [6]. Estimates for Sweden lie around 2% [4], whereas the prevalence in Denmark is twice as high for multiparous (3.6%) as for primiparous women (1.3%) [7]. A Swedish registry-based study showed that although caesarean section on maternal request (CSMR) increased three-fold over a 10-year period, it was a minor contribution to the overall rise in CSs [4].

Cesarean preference is strongly associated with fear of birth, previous CS and previous negative birth experience compared to women with preference for vaginal delivery [8, 9]. Women who prefer CS more often have characteristics such as higher age, low education level, unemployed, non-native origin, smoking, symptoms of depression and history of abuse [8–10]. Moreover, a cesarean preference is predictive of both planned and emergency CS outcomes [6, 8]. First-time women requesting planned CS do not always present with a clinically significant fear of childbirth, but have more negative expectations of vaginal delivery compared to women planning for vaginal delivery [11]. A qualitative study from Sweden showed that primiparous women requesting cesarean section often expressed deeply rooted emotions about natural birth since early adulthood [12]. Reasons reported among 91 Swedish women requesting CS in first pregnancy were fear of birth, safety issues, birth history of relatives, fear of pain and history of sexual abuse [13]. Parity may be crucial for understanding maternal requests, but few studies have shown stratified results for multiparous women. A higher prevalence among multiparas seems to be due to factors like previous cesarean or fear of birth rather than parity per se [9]. Understanding how and why the fear of giving birth increases with parity among some women is important for developing future care.

In contrast to a lively debate about maternal autonomy, there has been little discussion about reasons for CS requests and possible prevention and treatment strategies [14, 15]. Researchers have called for qualitative research on the subject to facilitate better understanding of cultural and psychosocial factors influencing maternal requests for CS in order to improve care for these women [16, 17]. Many qualitative studies have focused so far on primiparous women [18–20]. Women requesting CS is a diverse group of women for whom factors relating to parity may be important for understanding its sociocultural drivers. As the first purely qualitative study in Europe to include multiparous and primiparous women and their caregivers, our aim is to provide an in-depth exploration of women’s reasons for requesting a planned CS in Norway, in the absence of obstetric indications.

### Norwegian birth context

Primary care midwives and general practitioners (GPs) have the main responsibility for follow-up and care during pregnancy in Norway, while births and postnatal care are provided at public hospitals. There is no private delivery option, and all care during pregnancy is provided free of charge. Delivery care is primarily midwife-led, assisted by obstetricians in the event of complication. If a woman requests a planned CS, she must be referred for counseling at the hospital where she plans to give birth. Birth counseling at each individual delivery unit may be provided by a midwife or an obstetrician. Planned CS is officially not available on request, and considered only as indicated by an obstetrician [21]. For non-Norwegian and English-speaking women, interpreting services are provided if possible. The Norwegian CS rate was 16% in 2017 [22], a low rate as compared to other high-income countries. The county variance in CS rates in Norway ranged from 11.5–21.0% [22].

## Methods

A descriptive qualitative design was chosen to explore the research question in depth and to facilitate new understanding and knowledge. The study was undertaken at a university hospital in Norway with 5000 annual deliveries and a regional CS rate of 12.6% [22]. Women requesting CS were referred for birth counseling by their general practitioner or primary care midwife, and were seen at a midwife-led counseling center at the hospital. Internal referrals by obstetricians and midwives within the hospital also occurred. The final decision on a planned CS after the counseling process was taken by direct consultation with an obstetrician or by the midwife in charge of counseling after agreement with an obstetrician.

### Recruitment and data collection

Women were recruited consecutively for semi-structured in-depth interviews by midwives responsible for birth counseling at the hospital. Written information and an invitation to participate in the study were provided by the midwife if a woman was above 16 years of age, had presented an oral request for CS and had a normal pregnancy with no significant medical risk (interpreted as no obstetric indications for a planned CS). Informed consent was obtained prior to the study interview by the recruiting midwife or the first author. The women were interviewed late in their pregnancy or after birth. The interviews took place in the first author’s office or in the informant’s home if preferred. One woman was interviewed twice due to subsequent relevant information acquired late in her pregnancy. In-depth interviews were chosen to facilitate dialogue about this personal and sensitive issue. The interviews often took a narrative style and were opened with, “Would you like to tell me your story about why you want a planned C-section?”, followed by questions and probes from the interview-guide when needed (Additional file 1). Interviews usually appeared to be lively and self-driven reflecting that the women wanted to tell their story. Three informants were immigrants, born outside Norway, and were somewhat constrained by not being able to explain themselves in their mother tongue. Two of these women were interviewed in Norwegian and one in English.

A purposive sample of midwives (working in counseling, delivery and postnatal care) and obstetricians was selected to participate in focus group discussions consisting of three to four participants grouped by profession. Groups of three to four participants were chosen for primarily pragmatic reasons, but eventually experienced as a favorable size to facilitate discussion and allow participation by all informants. The focus groups were held at the hospital and selected to allow interaction and sharing of experiences and opinions between colleagues. In all focus groups, the conversations were lively and driven mainly by the participants, sharing positive and negative experiences and conflicting opinions. Questions from the interview guide for the focus groups are provided in the Additional file 2.

All interviews were carried out by the first author between June 2016 and August 2017. Focus groups were conducted during March and April 2017. An interview guide with open-ended questions was developed by the research team and modified during the process. Individual interviews lasted from 51 to 79 min; focus groups from 40 to 70 min. All material was audio recorded and transcribed verbatim by the interviewer. After 12 individual interviews and six focus groups, the material was evaluated as being sufficiently rich to illuminate the research question. Scheduled interviews were conducted and further recruitment ceased.

### Analysis

**Fig. 1:**
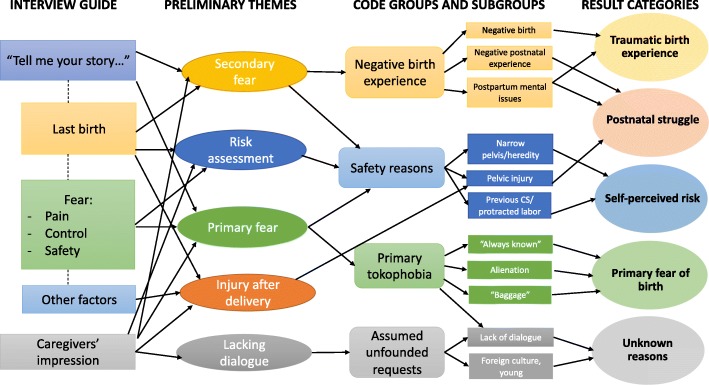
Illustration of the flexible analytic process with development of the main result categories (to the right). Figure modified with permission from Malterud [23]

The transcripts were organized with the coding software NVivo, version 11. Data were analyzed using Systematic Text Condensation [23, 24], a method for cross-case thematic analysis conducted in four steps: 1) Reading the transcripts stepwise during data collection to adjust the interview guide, evaluate saturation and identify main themes for further analysis. 2) Identifying meaning units in the text and coding into main groups. 3) Condensation by splitting into subgroups. 4) Synthesizing condensates into re-conceptualized descriptions. The first step was conducted by the first and last authors, steps 2–3 by the first author and step 4 by collaboration among all authors in a step-by-step process of discussion and reflection. The transcripts were analyzed using editing analysis style, drawing categories upon the empirical data rather than a theory-driven template analysis, although the analysis was influenced by existing empirical knowledge ([23], 95, p.). Data from women and caregivers were synthesized to inform the analysis, with findings among women supported and complemented by the experiences and impressions stated by caregivers. Figure 1 shows the analytic process, illustrating keywords and flexibility in the development of themes and coding of data into code groups and categories. Norwegian quotes have been translated into English by a Norwegian and English-speaking professional language editor and translator and back checked by the research team. Informants were coded numerically starting with W for woman, M for midwife and O for obstetrician.

## Results

### Participants

Seventeen women referred to the delivery unit for birth counseling with a cesarean request were interviewed. Women’s ages at the time of the interview ranged from 27 to 42 years. Fourteen women were multiparous women who had not requested a cesarean section in a previous pregnancy. Two women were second-time pregnancies and had been referred for birth counseling due to a cesarean request in both the current and a previous pregnancy. These women were interviewed about their rationale for cesarean preference during both pregnancies. One woman was pregnant for the first time and referred due to a cesarean request. Toward the end of pregnancy, 10 women were scheduled for a planned CS, while seven women planned for a vaginal delivery. Nine midwives and 11 obstetricians (six consultants, five residents) with varying lengths of experience were also interviewed in the focus groups. Their experience with and impression of the maternal group supported and complemented the findings from the women. Additional characteristics of the informants are presented in Tables 1 and 2 and indicate a favorably heterogeneous sample.

**Table 1:** Women’s characteristics

Characteristics	Number
N=	17
Age	
27–42	17
Relationship status	
Married	11
Cohabitating	5
Single	1
Education completed	
High school level	4
Bachelor’s level	7
Master’s level	6

**Table 2:** Caregivers’ characteristics

Characteristics	Number
N=	20
Profession	
Midwife	9
Obstetrician	11
Gender	
Female	16
Male	4
Age	
29–39	7
40–49	6
≥ 50	7
Years of experience	
≤ 5	5
6–10	6
11–20	5
> 20	4

### Main categories

Five principal categories emerged from the analysis of women’s rationales for requesting CS (Fig. 1). For some, fear of birth emerged as a consequence of a previous traumatic birth, and resulted in a preference for a planned CS as a way of avoiding repeated trauma. For others, negative experiences in postnatal care and the puerperal period led to a request for planned CS in order to ensure mental rather than physical capacity to care for the expected child. Some women were under the impression that they were at high risk of emergency CS, and requested a planned CS on the basis of self-perceived risk. Requests for planned CS in first pregnancies were based on deeply held fear accompanied by alienation towards the idea of giving birth. Additionally, obstetricians reported on experiencing requests without what they regarded as well-grounded reasons or significant fear.

### Previous traumatic birth experience: ‘Back on that butcher’s bench’

A previous traumatic birth experience typically encompassed multiple dimensions. A secondary fear of giving birth may arise shortly after delivery or during the next pregnancy. An important dimension of the previous birth experience was having experienced extreme fear during delivery, often involving a woman’s conviction that her own or the baby’s life was at risk. This could be due to dramatic events or insufficient information and support during or after delivery. Language barriers were a particular source of fear for immigrants, like the case of this woman born outside Norway:

“And I was so scared that I just understood the pulses [fetal heart rate] were going down, and I was scared like if my baby will come out alive, or it will be dead. And then I was just asking them, will she be alive? And nobody bothered to answer me.” W9 Multiparous woman

Midwives acknowledged the difficult balance of providing enough information without creating unnecessary fear. Communication between caregivers was also described as giving rise to fear and misunderstanding:

“They can retell almost verbatim how a conversation has occurred between two parties in a delivery room, and was almost perceived as warfare. Disagreement between midwife and doctor, which is perceived as enormous, creates a terrible insecurity for the woman.” M1 Midwife

Recalling extreme pain and lack of control was important in reluctance toward another attempt of a vaginal delivery, the one leading to the other:

“So in a way it’s the pain that was the reason. But then it wasn’t. Because the pain made me lose control … And when I lost control I panicked. And the panic made me go completely irrational. Then there was no way back … Then I remember I told him [partner] I am fainting now, you will just have to get that baby out.” W16 Multiparous woman

Operative delivery was a significant contributor to lack of control for some. Several women had experienced multiple vacuum and/or forceps attempts provided with either too much or too little analgesia (according to the women), the former leading to lack of control and the latter leading to extreme pain and shock, as in the case of this woman who explained that she failed to receive a pudendal block:

“They made it on the 4th [forceps] attempt. But by then I thought I was dead a long time ago. I had no clue what happened… They didn’t have time to pay any attention to me when she [the baby] was in bad shape, I get that. But one of the two [caregivers] present had had time to give me that [pudendal] analgesia… And I am a bit scared that even though it is all normal now. When the baby comes, I will go straight back. In my head I’m back on that butcher’s bench.” W13 Multiparous woman

A negative birth experience may result in distrust of the clinic. Some women were left with the impression of being subjected to mistakes, inadequate care or pain relief, experimental medicine, poor communication or being turned into a teaching case. One woman felt like a scientific case presented to a broad collegium in the delivery room. For some, an emergency CS felt like a relief when caregivers verbally summarized what they were about to do. A woman born outside Norway explained how lack of information and predictability left her with the impression of delivery care as “floating” and based on experiment rather than medical judgment:

“Like no one was sure what are they doing. Everyone, like how the situation is in villages in my home country, like ok now we will try this thing now we will try this thing, it was not like medically.” W9 Multiparous woman

Several women described being reluctant to become pregnant again after the last birth experience, delaying a new pregnancy for many years, becoming pregnant unwillingly or having received assurance of a planned CS prior to getting pregnant.

Many women expressed a need for, as well as an expectation of, some follow-up by the clinic after birth, especially after operative deliveries. All women in Norway receive a check-up free of charge with their GP 6 weeks postpartum, but there is no official provision of follow-up at the delivery clinics after discharge. Caregivers working at the hospital, however, provided targeted follow up in special cases, especially after obstetrician-assisted deliveries, consisting of either an in-house talk at the postnatal ward before discharge or by calling them in several weeks postpartum for a debriefing in the out-patient clinic. Most women had received a visit from the obstetrician responsible for their birth before discharge, but a few had not had that opportunity. The optimal timing suggested by the women for a postpartum talk was between three and 6 weeks postpartum; by that time the mother would have had time to adjust to her new situation and reflect on what had actually happened. Women preferred the postpartum talk to take place within a specialized care setting, rather than in primary care, and with caregivers who had insight into the clinic’s routines and delivery care.

“And if I had been sent to the right people straight after the birth the last time, then I wouldn’t necessarily have, first, refused to have [more] children, and second, when I finally had got it [pregnant], to be so scared of a potential birth. I feel that there is a lot that could have been avoided. If they would just have followed up properly… The baby survived, the mother survived. That’s good. But it’s not good enough.” W13 Multiparous woman

Caregivers also emphasized the importance of a postpartum follow-up appointment after birth. This would be an opportunity for debriefing, answering questions and clearing up misunderstandings. This was practiced to some extent by several of the caregivers, but not established as a routine. The main challenge was to identify the women that needed such follow-up. There were routines for a short in-house postpartum visit soon after all obstetrician-attended deliveries. The timing of this was regarded as suboptimal, providing the women with too little time to process the event and reflect on any questions she might have.

“... where I worked before I always had postpartum talks with these (women), I always saw them in the out-patient clinic after six weeks for a talk. And I believe I prevented a lot of fear of birth.” O6 Obstetrician

### Postnatal struggle: ‘I’d rather be present in my head than in my body'

The time following delivery was the crucial part of previous birth experiences for some women. Several women (and some partners, according to the women) had experienced difficulties processing the event, including shock, repression, depressive or anxious symptoms, but few had sought professional support. The experience of feeling mentally incapable of caring for the child due to difficulties with processing the birth experience was especially challenging. Some midwives emphasized how a negative birth experience can be exacerbated by negative experiences in the puerperium, such as feeling a lack of support, feeling incapable of caring for their child, and other difficult emotions following birth:

“I agree that a lot of what we see is that trauma often comes after delivery. The trauma comes from bad experiences in postnatal care… Several, we can see in hindsight, have been through a postpartum depression without receiving care. And it is a black hole. And it creates fear, for some a fear of dying.” M1 Midwife

Several women complained about lack of staff and support in the postnatal ward. They often felt too sick to care for their newborn properly after the birth experience, especially if their partner was sent home. Feelings of a lack of safety and being left on their own at the clinic made some demand early discharge against the clinic’s advice:

“I have never felt so unsafe and helpless as I was at the clinic… It took all my effort to pretend that I was well so that I got out of that madhouse.” W8 Multiparous woman

These women usually had experienced a protracted labor, emergency CS or operative delivery, and a planned CS was perceived to provide better health and an easier time after birth as compared to a complicated vaginal delivery.

Some women had experienced pelvic complications (urinary/anal tract damage, chronic pain) followed by handicap, social stigma and frustration in getting help. While afraid of aggravating the present injury by another vaginal delivery, these women also regarded a planned CS as a way of avoiding recurrence of a difficult time following birth, as in the case of this woman who had experienced a pelvic floor injury with urinary incontinence leading to a difficult time after delivery, both emotionally and socially.

“Some are just a bit unlucky, and things happen during the birth. And unfortunately, I was one of them. And that’s why I’m thinking that I don’t want to (give birth). I am terrified of it happening again.” W5 Multiparous woman

Overall, many women in this category regarded a planned CS as a predictable and calm birth experience that in turn would facilitate a mentally stable puerperal period. The anticipated mental benefit after a planned CS was worth the longer recovery time in physical terms, as described by this mother expecting her second child:

“Because now I have two small children to think of. Then I’d rather be present in my head than in my body.” W1 Multiparous woman

### Fear due to safety reasons based on self-perceived risk: ‘I can’t give birth normally, I’m convinced'

Several women based their request on what they personally considered medical risk factors. They were concerned about complicated births running in their families, previous protracted labor/emergency CS, perception of having a narrow pelvis or expecting a big baby. While some were afraid of experiencing stillbirth, others simply wanted to avoid a stressful emergency situation. The conviction of not being able to deliver vaginally was recurring:

“I hear from my family that my great-grandmother and great-great-grandmother had lots of stillbirths because they [the unborn child] got stuck… And my maternal grandmother had a C-section with my mum, and my mum had a C-section with me… So, I can’t give birth normally, I am convinced of it.” W8 Multiparous woman

Caregivers were aware of these requests, although they might disagree about the medical significance of the upcoming birth. The women’s rationale was to avoid an emergency CS by having a planned one, as in this case of a woman who had reviewed the academic literature on her own:

“I checked the guidelines of the Royal College of Obstetricians and Gynecologists… They concluded in the end that [for women with previous CS] the overall risk of a vaginal birth was quite small, but still safer with a C-section… And if you have a big baby, the risk of having a C-section is already 50%… It’s the emergency in this I want to avoid.” – W17 Multiparous woman

### Primary fear of birth: ‘It’s just an anxiety that I have'

Some women presented with deeply rooted fear they had carried since their early teens, making them feel different from other people. It was experienced as an encompassing primary phobia that had accelerated with pregnancy. They had typically delayed becoming pregnant. One of the women based her fear on a traumatic experience in her early youth giving rise to a fear of death during delivery.

“I’ve been frightened. I thought it might change, go away some of the time during the pregnancy, but it didn’t go. The closer I got the more scared I was… Death and pain, they were the only things in my head.” W3 Primiparous woman

Another woman was not able to describe properly what her fear was about; it was a deeply rooted feeling of birth being completely unnatural to her:

“I cannot understand what makes me so different from others. And that has perhaps been partly what’s been the most unpleasant, to feel that it is experienced as different. Because it is in a way something all women are supposed to feel as a natural part of life. But I don’t believe it is anymore natural than for a man, without that making me different. It’s just an anxiety that I have.” W6 Multiparous woman, requesting CS in her first and the current pregnancy

According to caregivers, these women could sometimes be extremely scared and difficult to convince about a vaginal delivery. They emphasized how some were particularly vulnerable and carried “excess baggage” from earlier life. Some had experienced sexual assaults or other traumatic life events that, one midwife underscored, would not always be revealed during counseling. They often carried a sense of alienation toward giving birth and having children in the first place:

“Some have a psychological baggage from earlier in life, and have perhaps delayed becoming pregnant, are scared of being so and of having a child at all. Birth is very strange to them… They don’t believe they will cope with it.” O11 Obstetrician

### Requests based on unknown reasons – lack of dialogue

Obstetricians were especially concerned with a minority of women requesting CS who presented without well-grounded reasons or significant anxiety. Willingness to comply with such requests was lower, and willingness to spend time and effort on them varied. Such requests were rare, and it was difficult to obtain a good dialogue. These women could be very determined about their choice of delivery; they were sometimes very young and possibly without understanding about the implications of surgery.

“And there is something about those who you absolutely do not get into dialogue with, who just sit there and say no no no. Won’t have a story, won’t have a background… And sometimes they are very young. Who absolutely do not understand this. Who just think it is much easier with surgery and then finished” O1 Obstetrician

Sometimes these were women immigrated from countries with high C-section rates:

“Those cases that are not anxiety for birth are those who have seen in the media, heard from friends, read and think, ‘Oh what an easy solution to have C-section’… You have the normal birth which most people have to accept, and you have the Hollywood version where you’re admitted to the hospital and get a planned C-section, free from perineal tears, baby comes out newly washed. That’s not a medical indication. I had one patient from abroad with that kind of argument. From a country with a very high C-section rate.” O5 Obstetrician

## Discussion

Although previous birth experiences are central to many, there are nuanced reasons and various rationales behind a cesarean request. Traumatic birth or postnatal experiences were important for some women, whereas others based their request on self-perceived risk. Primary fear of birth in first pregnancies also appeared, as did ‘requests based on unknown reasons’, according to obstetricians. This multifactorial complexity behind maternal requests is in accordance with the findings of other qualitative studies [25].

Background characteristics of women requesting C-sections suggest a population susceptible to mental illness [8, 10, 26]. The rationale for many women was to avoid mental health problems following a traumatic birth experience. However, little is known about the impact of a planned CS on mental health after birth. Provision of a planned CS does not seem to lower antepartum anxiety or depressive symptoms, but to compel a vaginal delivery may lead to post-traumatic stress and depression [27]. To be able to give proper recommendations during birth counseling for women with a fear of birth, mental health has to be addressed in the trade-off between risk and benefits with regard to delivery mode.

Fear of birth due to previous birth experience was the dominant reason for cesarean requests among the women in this study. Twelve out of 14 multiparous women had experienced either operative delivery and/or emergency CS. Other studies have shown strong associations between cesarean preference and previous negative birth experience, previous CS and fear of birth [6, 8, 26]. Our findings suggest that it is not the previous CS but rather the negative aspects of the birth experiences, which are crucial in their justification of a cesarean request. This is in line with a systematic review of qualitative literature also reporting previous birth experiences as an important reason for requesting CS [25]. Størksen et al. found that 8% of pregnant women in Norway had significant fear of birth, which was highly predictive of a cesarean preference. Presence of a previous negative birth experience was the strongest predictor of fear, followed by impaired mental health and lack of social support. Only 13% of women with fear received a CS, and very few requested CS in the absence of a previous negative birth experience [26]. If various traumas from a previous birth experience are the major causes of CSMR, we should acknowledge the phenomenon as partly an iatrogenic problem. Fear of birth due to previous traumatic birth experience can be prevented through proper midwifery and perinatal mental health care [28]. Women and caregivers interviewed in this study suggested postpartum follow-up after birth as a way of avoiding cesarean requests in subsequent pregnancies. A challenge described by caregivers was how to capture the subjective trauma. A subjective negative birth experience is not necessarily determined by an obstetric event, but rather by lack of support and poor-quality care during childbirth [29, 30]. Prevention and follow up must therefore be targeted. Whether postnatal debriefing improves postpartum mental health and avoids development of post-traumatic stress is currently uncertain [31]. Women seem to appreciate such services and midwives regard it as beneficial for women [32].

Several women (including one woman who had requested CS in her first pregnancy) based their request on safety reasons due to self-perceived risk for, and as a means of avoiding, an emergency CS. Previous birth experience, if present, was not necessarily described as negative or traumatic. This may partly explain why clinical anxiety is not present in all women requesting CS [11]. Several researchers have highlighted the association between CSMR and previous or current obstetric complications, bringing maternal perception of risk into account [33–35]. These requests may call for a different healthcare response than the more trauma-based requests. Over all, adequate healthcare toward this patient population seems to necessitate targeted approaches.

A study among primiparous women from Sweden revealed how deeply rooted emotions beyond fear of birth dominated their requests for a planned CS [19]. As for two out of three women having requested cesarean section in their first pregnancy in our study, these women described that they had always known they would not wish to give birth the natural way. According to caregivers, primiparous requests of this kind were not usual. Others have indicated that several women with fear of childbirth do not accept psychological counseling and demand a CS without further discussion [36–38]. Obstetricians were especially concerned with the rare but present women who gave no well-grounded reasons for their request and had a lack of willingness to establish a dialogue. Midwives did not discuss these women and may have achieved a better dialogue and understanding of these requests. Nevertheless, we were not able to probe specifically on this issue during data collection since the midwives working in counseling care were the first focus group interviewed.

### Strengths and limitations

The majority of women interviewed in this study gave birth by a planned CS at a hospital with a low CS rate compared to the overall country. Midwives at the counseling center suggested that approximately 70% of women withdrew their request during birth counseling. This may imply that the women in this study already had a strong motivation for planned CS, and may partly explain why requests based on unknown reasons were not represented among these women. The female interviewer had no direct relation to the clinic. This opened up an honest dialogue and promotes the credibility of the findings. The majority of women were multiparous women with a previous experience of attempted vaginal delivery. Inclusion of only three women having requested CS in their first pregnancy may question the credibility of findings regarding first-pregnancy requests. However, our findings from the focus groups with caregivers and existing literature support these results. Most women were interviewed late in their current pregnancy, and some women were interviewed after giving birth. Two second pregnancy women requesting CS in both their current and the previous pregnancy were interviewed regarding their rationale for requesting CS in both pregnancies. Recalling prenatal fear and other factors influencing their wish may have been affected by the birth experience and memory at the time of the interview, which may have influenced the results. However, postpartum interviews also provided the advantage of illuminating the phenomena from a comprehensive pre- and postpartum perspective, and all women provided spontaneous and rich descriptions about their reasons and rationales for wanting a planned CS. There was an overall strong interview dialogue with lively and self-driven discussion pointing toward high information power [39]. The heterogeneous characteristics of informants (Tables 1 and 2) and the complementation of women’s and caregivers’ perspectives add credibility to the study. As a multidisciplinary research team (i.e., a newly educated medical doctor, an experienced obstetrician and a philosopher), we were able to approach and discuss the research question and findings from different angles and ensure consistency in the analysis. Even though the study is constrained by the setting, birth and fear relate fundamentally to the human condition, permitting transferability to other settings as well.

## Conclusion

Cesarean requests are based on varying rationales and life experiences. Previous birth experience occurs as a major driver of subsequent fear of birth. Thus, CSMR should be regarded partly as an iatrogenic problem with potential for improvement and prevention both during and after deliveries. Some women based their requests on concerns about a perceived high risk of emergency CS, which may call for a different counseling approach. Over all, prevention and healthcare should be carefully targeted according to such findings.

## Additional files

Additional file 1: Interview guide for in-depth interviews with women. Questions and probes used in interviews with women. (DOCX 15 kb)

Additional file 2: Interview guide for focus group discussions with caregivers. Questions and probes used in focus group discussions with caregivers. (DOCX 14 kb)

## Abbreviations

CS: Cesarean section
CSMR: Cesarean section on maternal request
