# Supplementary material for: Maternal reasons for requesting planned cesarean section in Norway: a qualitative study
Source: BMC Pregnancy Childbirth. 2019 Mar 29;19:102. doi: 10.1186/s12884-019-2250-6 (PMC6440101; doi:10.1186/s12884-019-2250-6)
Supplement: Supplementary file 1 — Interview guide for in-depth interviews with women. Questions and probes used in interviews with women. (DOCX 15 kb) [file 12884_2019_2250_MOESM1_ESM.docx]

Additional file 1

| No. | Question | Probes |
| --- | --- | --- |
| 1. | Would you like to tell me your story about why you are requesting a C-section? |  |
| 2. | What is the reason for your wish for a C-section? | - If previous birth experience, would you like to tell me about it? - What do you fear?   - Pain?   - Control?   - Injury to yourself or the child? - Other reasons? - Something special that may have influenced your choice or attitude toward this? |
| 3 | How has it been to relate other people about your wish for a cesarean section? | - Have you been able to confide in someone? - Do you have someone to talk to? - Do you wish to talk to others about it? (E.g. partner, family, friends?) |
| 4 | What kind of information have you received or sought for yourself? | - Satisfied with the information? - Sought information on your own? - Where? Sources? Critical? |
| 5 | How have you proceeded to get help with your cesarean request? | - Conversations with GP/midwife in primary care? - Referral to specialized care? |
| 6 | Tell me about your experience with counseling. | - Expectations, information, experience |
| 7 | Is there anything that could have been improved for you or others in your situation? |  |
| 8 | Who do you believe should make the final choice of delivery mode? |  |
| 9 | Is there anything else you think I should know? |  |

**Interview guide for in-depth interviews with women**
