# Supplementary material for: Maternal reasons for requesting planned cesarean section in Norway: a qualitative study
Source: BMC Pregnancy Childbirth. 2019 Mar 29;19:102. doi: 10.1186/s12884-019-2250-6 (PMC6440101; doi:10.1186/s12884-019-2250-6)
Supplement: Supplementary file 2 — Interview guide for focus group discussions with caregivers. Questions and probes used in focus group discussions with caregivers. (DOCX 14 kb) [file 12884_2019_2250_MOESM2_ESM.docx]

Additional file 2

**Interview guide for focus group discussions with caregivers**

| No. | Question | Probes |
| --- | --- | --- |
| 1 | What is your impression of women who request cesarean? | - Who are they? - Why do they want C-section? |
| 2 | How is it to work with these patients? | - What kind of emotions do they evoke? - Do you experience any ethical challenges facing them? |
| 3 | Would you like to tell me about how you handle these patients? | - Strategies? Improvements? |
| 4 | How do you think the decision should be made? Who should make the final choice? | - Shared, doctor, midwife, the woman? |
